# Vulvovaginal *Candida* *albicans* Clinical Isolates’ Resistance to Phagocytosis In-Vitro

**DOI:** 10.3390/life12060838

**Published:** 2022-06-04

**Authors:** Paula Faria-Gonçalves, Ana Sofia Oliveira, Carlos Gaspar, Lisa Rodrigues, Rita Palmeira-de-Oliveira, José Martinez-de-Oliveira, Teresa Gonçalves, Ana Palmeira-de-Oliveira, Joana Rolo

**Affiliations:** 1 CICS-UBI—Health Sciences Research Center, University of Beira Interior, 6200-506 Covilhã, Portugal; paula.goncalves@ubi.pt (P.F.-G.); ana_g2s@hotmail.com (A.S.O.); cgaspar@fcsaude.ubi.pt (C.G.); rpo@fcsaude.ubi.pt (R.P.-d.-O.); jmo@fcsaude.ubi.pt (J.M.-d.-O.); apo@fcsaude.ubi.pt (A.P.-d.-O.); 2 FCS-UBI—Faculty of Health Sciences, University of Beira Interior, 6200-506 Covilhã, Portugal; 3 FMUMN—Faculty of Medicine, University Mandume Ya Ndemufayo, Lubango 3FJP+27X, Angola; 4 Labfit-HPRD—Health Products Research and Development Lda, 6200-284 Covilhã, Portugal; 5 CNC—Center for Neuroscience and Cell Biology, University of Coimbra, 3004-504 Coimbra, Portugal; lisa.rodrigues@uc.pt (L.R.); tgoncalves@fmed.uc.pt (T.G.); 6 FMUC—Faculty of Medicine, University of Coimbra, 3004-504 Coimbra, Portugal

**Keywords:** adhesion, macrophages, virulence, vulvovaginal, yeast

## Abstract

Previous studies have revealed that *Candida albicans* isolates involved in chronic vulvovaginal candidosis (cVVC) phenotypically express less virulent traits than clinical isolates involved in sporadic infections. In this study, we aimed to further explore this finding by studying the behaviour of those same clinical isolates in in-vitro models of infection. Eighteen clinical *Candida albicans* isolates were collected from women suffering sporadic (eight isolates) or chronic infections (ten isolates). Adhesion to HeLa cells (human cervical cancer epithelial cell line) and resistance to phagocytosis by RAW 264.7 cells (murine macrophages cell line) were tested in-vitro. In addition, phenotypic expression of virulence factors related with either adhesion or resistance to phagocytosis was tested in-vitro. Results indicated that yeast isolates involved in sporadic infection adhered in a higher proportion of HeLa cells than those of chronic infections, which was related with their ability to produce biofilm (*p* < 0.05). The ability to evade phagocytosis was related to an elevated production of proteases (*p* < 0.05) by chronic isolates, while sporadic isolates’ resistance to phagocytosis was related to a higher hydrophobicity of cell walls (*p* < 0.05). We conclude that the evasion of macrophage-mediated phagocytosis related to the production of proteases might be an important factor involved in the recurrence of vulvovaginal candidosis infection.

## 1. Introduction

Vulvovaginal candidosis (VVC) is a disease affecting millions of women worldwide [1]. It is most frequently caused by *Candida albicans*, a dimorphic yeast [2]. Other species of *Candida* can also cause this affliction, namely *Candida glabrata* [3]. VVC is characterized by an overgrowth of yeast in the vaginal mucosa, leading to swelling, irritation, and excessive vaginal discharge. *Candida* spp.’ infection of the vaginal mucosa has been related to its capacity to form biofilms [4], to produce germ tubes (filamentation), and to produce extracellular enzymes such as phospholipase and proteases [5]. In addition, evasion of the host immune system and consequent inflammation has also been identified as a virulence factor contributing to *Candida* spp. pathogenesis in this specific niche. Particularly, epithelial invasion and immune cell infiltration have been found to be key developments for the progression of the disease. Neutrophil recruitment has been related to the onset of symptomatology [6], particularly for *C. albicans*, which appears to be especially vaginopathic [7], due in part to its ability to produce hyphae and to express candidalysin, a potent exotoxin [7,8]. 

Re-infection of the vaginal mucosa can occur, a condition commonly known as recurrent vulvovaginal candidosis or chronic candidosis (cVVC). cVVC is characterized by the occurrence of 3 or more episodes in the period of one year [9], causing high morbidity. Several risk factors have been described as contributors to recurrence such as pregnancy and/or diabetes mellitus. However, apart from the increase in immune cell recruitment already reported [6], the cues used by *Candida* spp. to persist in the vaginal mucosa are largely elusive. Our previous studies have identified that chronic *Candida* isolates are weak biofilm and germ tube producers, when compared with isolates involved in sporadic infections [10]. A recent study has revealed that vaginal isolates show adaptative behavior in their interaction with macrophages in-vitro, reflecting their infective ability [11]. In this work, we aim to further explore differences in the immunopathogenicity of *Candida albicans* isolates involved in cVVC by studying their interaction with epithelial and macrophage cell lines.

## 2. Materials and Methods

### 2.1. Yeast Isolates

Eighteen vulvovaginal candidosis isolates were included in the study. The isolates were obtained from 15 women attending a gynaecological consultation at a private clinic. The study was approved by the Ethics Committee of the research centre where the experiments were conducted, CICS-UBI (approval CE-UBI-Pj-2018-022). The strains were described in previous studies, in regard to their susceptibility to fluconazole and ability to form biofilms [3,10] (Table 1). The identification of the *Candida albicans* isolates was obtained using gold-standard techniques routinely used for identification in clinical laboratories. A representative collection out of the collections used in previous studies was used in this study, in order to include *C. albicans* isolates with different phenotypic profiles. As recently reviewed in [5], in this study, chronic VVC (cVVC was characterized by the occurrence of 3 or more episodes in the period of one year. Sporadic VVC (sVVC) was defined as the occurrence of a single episode of VVC infection in a period of one year. A *C. albicans* type strain, ATCC 10231, was used in all experiments as internal control. 

### 2.2. Determination of the Ability to Produce Proteinase

The production of proteinases was evaluated with the procedure described in Raja Vinodhini et al., 2016 [12]. A cell suspension was prepared in sterile PBS at 0.5 MacFarland and inoculated (10 µL) in proteinase agar plates containing 2% glucose, 0.1% KH_2_PO_4_, 0.05% MgSO_4_, 2% agar, and 1% BSA solution in distilled water. The plates were incubated for 10 days at 37 °C. The enzymatic activity (Pz) was determined by calculating the ratio between the diameters of the colony vs. the precipitation zone. According to the literature [12], the lower the Pz value, the higher the enzymatic activity. When Pz = 1.0, the enzymatic activity is absent; when 0.64 < Pz < 1.0, the enzyme activity is positive; and when Pz ≤ 0.63 there is a strong positive enzymatic activity. *C. albicans* ATCC 10231 was used in each assay as the positive control (Pz ≤ 0.63). Two independent assays were performed for each isolate, being the result only validated when the standard deviation between the results obtained in each assay was less than 0.1.

### 2.3. Cellular Surface Hydrophobicity

The comparative analysis of cell surface hydrophobicity (CSH) was determined as previously described [13]. A suspension of yeast cells was prepared in 10 mL of sterile PBS at 0.5 MacFarland, and distributed into three test tubes (1.3 mL each). Afterwards, 100 µL from each of these test tubes were distributed into 96 well microtiter plates and initial OD was read at 620 nm using a microplate reader (Anthos 2020 microplate reader, Bio-Rad, CA, USA). Then, 0.3 mL of xylene (VWR Chemicals) was added to the remaining cell suspension in the test tubes (1.2 mL), mixed vigorously for 3 min and allowed to separate for 15 min. A total of 100 µL of the lower aqueous phase was carefully added to the wells of the microtiter plate and final OD was read at 620 nm. Tubes without cells served as control. Triplicates were used for each sample and the experiment was repeated twice. The percentage of cell surface hydrophobicity was calculated by using the formula Percentage CSH = (1 − final OD of aqueous phase/initial OD of cell suspension) × 100; results were presented as percentage of CSH ± SD (standard deviation).

### 2.4. pH Survival Test

Cellular suspensions at 0.5 McFarland were prepared in 2 mL YPD (Fisher Bioreagent) and adjusted to pH 5, pH 7, and pH 9. To assess the number of colony-forming units (CFU)/mL of the initial suspensions, 20 µL of each of these suspensions were transferred in triplicate to a 96 well plate, serial diluted and plated in SDA. The remaining volume of the suspension was placed in a 10 mL sterile glass tube and incubated in the orbital shaker (Argitob 200 Aralab, Sintra, Portugal) at 37 °C for 24 h at 180 rpm. After that, the cultures at different pHs were removed and the number of surviving yeasts was estimated by plating aliquots of serially diluted cultures in SDA, and incubating at 37 °C for 48 h. The resulting CFU/mL at different pHs were compared with the initial suspension.

### 2.5. Cell Culture and Co-Culture Assays 

RAW 264.7 cells, a mouse leukaemic monocyte macrophage cell line from the European Collection of Authenticated Cell Cultures (ECACC, UK; Catalogue number 91062702), was included in this study. Cells were cultured in Dulbecco′s Modified Eagle′s Medium-DMEM (Gibco, Alfagene, Lisboa, Portugal) with 4.5 g/L of glucose (Sigma-Aldrich, St. Louis, MO, USA), and supplemented with 10% (*v*/*v*) of non-inactivated Fetal Bovine Serum-FBS (Gibco, Alfagene, Lisboa, Portugal), 100 U/mL penicillin, 100 μg/mL streptomycin (Gibco, Alfagene, Lisboa, Portugal), and 1.5 g/L of sodium bicarbonate (Sigma-Aldrich, St. Louis, MO, USA), at 37 °C in a humidified atmosphere of 95% air and 5% CO_2_. Sub-confluent cultures (~70–80%), were split using cell scrappers to remove attached cells, according to ECACC recommendations. RPMI 1640 medium (Sigma-Aldrich) supplemented with 10% inactivated FBS, 23.8 mM sodium bicarbonate and 50 mM glucose was used for the experimental assays. For the co-culture assays with RAW 264.7 cells, a previously optimized procedure was performed [14,15]. Briefly, 1 × 10^5^ cells *per* well were seeded in a 96-well plate and incubated for 18 h. Thereafter, the culture medium was removed and cells were washed twice with sterile pre-warmed PBS 1X. *Candida* suspensions prepared in RPMI were added to the cells (200 µL/well) in a multiplicity of infection (MOI) of 1 and incubated at 37 °C for a period of 3 h. To quantify the yeasts viability, supernatants were collected and subjected to serial 1:10 dilutions up to 10^−6^ in multiwell plates, in triplicate (containing viable yeast cells that were not internalized). After removing all the supernatant, the adhered macrophages were lysed by adding 200 µL of sterile MilliQ water supplemented with 50 µL of 0.5% triton X-100 (Fisher) to each well. The obtained cell suspensions were serially diluted and aliquots of all dilutions were plated in SDA (corresponding to internalized yeast cells). After 48 h of incubation, the colonies were counted to estimate the number of CFU/mL in both conditions. The cell line HeLa, derived from human cervical adenocarcinoma was obtained from the American Type Culture Collection (ATCC-LGC Promochem, Teddington, United Kingdom). These cells were cultured in DMEM-F12 medium (Gibco, Alfagene, Lisboa, Portugal), supplemented with 100 U/mL penicillin, 100 μg/mL streptomycin (Gibco, Alfagene, Lisboa, Portugal), 10% (*v*/*v*) of inactivated FBS (Gibco, Alfagene, Lisboa, Portugal) and 1.5 g/L of sodium bicarbonate (Sigma-Aldrich, St. Louis, MO, USA). Cells were subcultured when confluence reached 70% using Trypsin-EDTA (Gibco, Alfagene, Lisboa, Portugal). For the co-culture assays with HeLa cells, an adaptation of a previously described procedure was performed [16]. Briefly, a cellular suspension of *Candida* spp. prepared in DMEM-F12 culture medium was incubated with a layer of HeLa cells with a multiplicity of infection (MOI) of 10, during 2 h. After removal of the non-adherent yeast cells with PBS, the adherent yeast cells were estimated by plating an aliquot of Trypsin-EDTA-treated supernatants on SDA. Colonies were counted after 48 h of incubation. 

### 2.6. Statistical Analysis 

Differences between clinical groups (cVVC vs. sVVC) were assessed with the non-parametric Mann-Whitney test. In addition, the correlation between the parameters observed within each clinical group was determined using the Spearman test (one-tailed *p*-value). Differences were considered statistically significant when the *p*-value was below 0.05 (95% confidence interval).

## 3. Results

**Figure 1:**
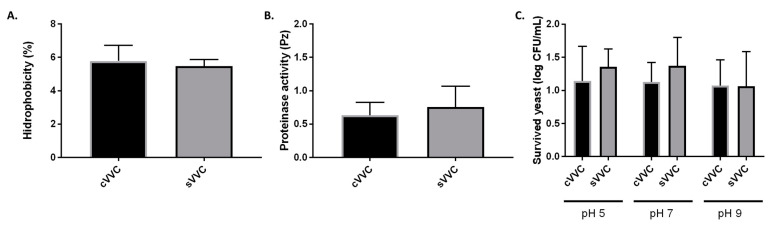
Phenotypic expression of virulence factors of the clinical isolates. (**A**) Hydrophobicity (%) of cell walls. (**B**) Proteinase activity (Pz value). (**C**) pH survival assay; the log difference (log CFU/mL) regarding the initial suspension is shown. In all assays, differences between clinical groups were found to be non-significant (*p* > 0.05). cVVC: chronic vulvovaginal candidosis isolates; sVVC: sporadic vulvovaginal candidosis isolates.

**Figure 2:**
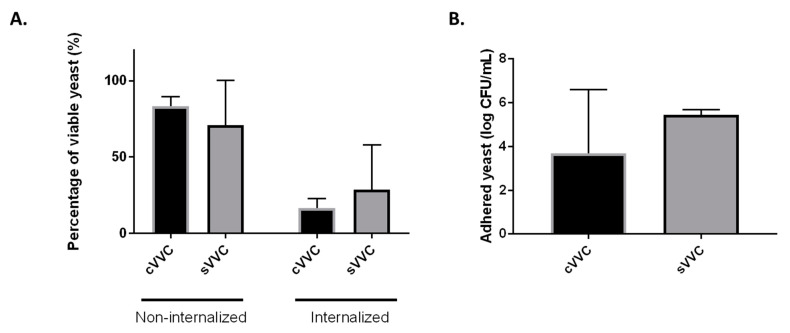
Interaction of clinical vulvovaginal isolates with cell lines. (**A**) Percentage (%) of non-internalized and internalized viable yeast cells, compared with the initial suspension, after infection of RAW 264.7 cells with an MOI of 1 during 3 h. For each isolate, three infections assays were performed (n = 3), and each in triplicate. (**B**) Number of adhered yeast cells (log CFU/mL) after infection of HeLa cells with an MOI of 10. In both RAW264.7 and HeLa assays, differences between the two clinical groups were found to be non-significant (*p* > 0.05). cVVC: chronic vulvovaginal candidosis isolates; sVVC: sporadic vulvovaginal candidosis isolates.

One of the main objectives of this work was to tackle differences between cVVC and sVVC in what regards phenotypes specifically described as determinants for virulence in *Candida albicans*. In this work, we studied the proteolytic activity, the cell wall hydrophobicity, and the ability to survive at different pH values. Overall, we found that cVVC isolates seemed to be more virulent than sVVC isolates as they produced more proteases (as noted by lower Pz values) and the hydrophobicity of their cell wall showed to be slightly higher (Figure 1A,B and Supplementary Figure S1). Regarding the pH survival test, we found that, at pH 9, cVVC isolates were more resistant, since cellular multiplication was not impaired at this condition (Figure 1C). The isolates belonging to the two clinical groups also behaved differently when in contact with cell lines. Specifically, sVVC isolates adhered better to Hela epithelial cells, as indicated by the higher number of CFU/mL that was recovered after the co-culture (Figure 2). Regarding the interaction with RAW 264.7 macrophages, we found that both cVVC isolates and sVVC isolates were able to escape phagocytosis, as evidenced by the high number of non-internalized yeasts (when compared with the initial suspension) and to remain viable even after internalization, although not to a great extent (Figure 2). However, we found that there were no statistical significant differences between the phenotypic expression of virulence factors among the two clinical groups (cVVC and sVVC; *p* > 0.05) (Figure 1), as well as the interaction with cell lines (*p* > 0.05). 

The correlation, for each clinical group, between the results obtained with the co-culture assays and the phenotypic expression of virulence factors was assessed using the Spearman test (Table 2). In regards to the assays with macrophages, we found that for the cVVC isolates there was a significant correlation (*p* < 0.05) between the percentage of viable internalized yeasts and proteinase activity. On the other hand, in the sVVC group, a significant correlation (*p* < 0.05) was observed between the proportion of viable internalized yeasts and the level of hydrophobicity of yeast cell walls. In the epithelial-adherence test (co-culture with HeLa cells), we found a significant correlation (*p* < 0.05) between the number of CFU/mL recovered after incubation and the ability to form biofilms in-vitro (as determined in Table 1 [10]) only for the sVVC clinical group (Table 2).

## 4. Discussion

In previous studies, we have found that sVVC isolates seemed to be more virulent, since their phenotypic expression of biofilms and filamentation was higher [10]. Therefore, we hypothesize if the success of repeated infection of cVVC isolates would be related with their increased ability to interact with the host’s cells. In this study, we found that the results obtained with cVVC isolates were not statistically significant, different from the results obtained with sVVC isolates, which illustrates the importance of these virulence factors to the immunopathogenicity of vulvovaginal *Candida albicans* isolates as a whole, as already described [5]. Recent reports focusing on isolates from different clinical origins involved in vulvovaginal candidosis reached the same conclusions [11,17].

One of the processes involved in phagocytosis mediated by macrophages is abrupt pH differences [18]. Resistance to pH differences can also be related with the increased ability of the strains to invade the vaginal mucosa. Therefore, we sought to determine if differences in the pH survival would be correlated with the ability to resist macrophages. We found no significant differences between the two clinical groups, or no relevant correlation (*p* > 0.05). Therefore, resistance to pH could be a virulence characteristic that is expressed by vulvovaginal *C. albicans* isolates as a whole and not particularly important for chronicity.

The ability to produce proteases by *C. albicans* has been linked with their virulence potential [19,20]. The secretion of aspartic proteases (SAP) has been implicated as a virulence factor leading to not only epithelial damage but also to inflammation [19,21]. Moreover, SAP have been also described as strong immunomodulators, independently of their proteolytic activity [19]. Therefore, to colonize and infect the vaginal niche, yeasts benefit from the phenotypic expression of this virulence factor [22], as illustrated by the high frequency of *SAP* genes found among isolates recovered from vaginal infections [23] and the fact that the genes coding for these enzymes are highly expressed during vaginal candidosis [24]. We did find a statistically significant correlation between the ability to evade phagocytosis and an elevated production of proteases of cVVC isolates. We hypothesize that strong protease producers such as cVVC isolates also resist better to macrophage attacks, as described early on [25]. 

On the other hand, sVVC isolates’ resistance to phagocytosis was related to a higher hydrophobicity of cell walls. The hydrophobicity of the cell wall is largely influenced by its composition. The composition of the *Candida* cell wall has been studied as a virulence factor. The cell wall is composed of glucose polymers 1,3 and 1,6 β-glucans, chitin, and mannosylated proteins. The differential expression and exposure of cell wall components are thought to be a major virulence factor influencing the pathogenicity of the yeast isolate [26,27]. In fact, hydrophobic cells are more adherent to epithelial tissues than hydrophilic cells [28]. However, other virulence factors can also be involved, such as the ability to form biofilm [4]. Therefore, our finding that sVVC isolates adhered in a higher proportion to HeLa cells (*p* < 0.05), together with their ability to produce biofilm already reported by our research group for these isolates [10], might be related to the higher hydrophobicity now registered in sVVC. *Candida* biofilms and their relationship with vulvovaginal candidosis have been extensively studied [4]. Previous results obtained by us indicated that sVVC isolates were strong biofilm producers, compared with cVVC isolates [10], which is in accordance with these results.

In this study, we did not find a statistically significant difference between the two clinical groups concerning yeast-macrophage interaction, in accordance with a recent report [11] describing that resistance to phagocytosis is highly strain-specific. However, these same authors describe that sVVC isolates change the composition of their cell walls when grown in a vaginal simulating medium containing lactate [11]. We hypothesize that isolates involved in sporadic infection have a more hydrophobic cell wall and produce a stronger biofilm, in order to adhere to epithelial cells and escape phagocytosis. On the other hand, cVVC are less adherent but produce a larger amount of proteases, that would help to invade the tissue and lead to inflammation, a major factor contributing to the progression of the disease [6]. Therefore, targeted treatment strategies could be employed taking into account the mechanisms used by *C. albicans* to persist in the vaginal mucosa; particularly topical treatments related to protease inhibitors could be an option for complicated cVVC cases [29]. Nevertheless, more studies are needed to address this issue.

Previous studies have highlighted differences related to the ability to switch to the filamentous form observed in *C. albicans* and not other *Candida* species, a characteristic that has been linked with the high virulence of *C. albicans* [30]. In fact, evasion of phagocytosis mediated by macrophages has been linked with the formation of hyphae [31], although other reports have identified macrophage evasion independently of filamentation [32]. Other studies have highlighted the importance of the pseudohyphae in the stimulation of the inflammasome [33]. The persistence of *C. albicans* in the mucosa has been mostly linked with its ability to form biofilms and to resist azole treatment [34], but other unknown virulence factors could also be implicated. It would be interesting to test other virulence factors like the expression of candidalysin that have been implicated in the immunopathogenesis of VVC/cVVC [5]. Another important key aspect to test would be the quantification of cytokines by the cell lines that would lead to neutrophil infiltration [6], a key aspect for the progression of the disease.

We conclude that yeast strains involved in vulvovaginal candidosis showed an equal ability to evade macrophage-mediated phagocytosis and to adhere to epithelial cells. We also found that cVVC isolates´ability to evade macrophages might be related to the production of proteases, although further studies are needed to understand the role of this virulence factor in the recurrence of vulvovaginal candidosis.

## Figures and Tables

**Table 1:** Characterization of the collection of strains used, as reported in [3].

Isolate	Patient	Species	cVVC/sVVC	Fluconazole Susceptibility	Biofilm Formation
cVVC1	P1	*C. albicans*	cVVC	Resistant	Strongly adherent
cVVC2	P1	*C. albicans*	cVVC	Resistant	Strongly adherent
cVVC3	P1	*C. albicans*	cVVC	Resistant	Strongly adherent
cVVC4	P2	*C. albicans*	cVVC	Resistant	Strongly adherent
cVVC5	P3	*C. albicans*	cVVC	Resistant	Strongly adherent
cVVC6	P3	*C. albicans*	cVVC	Resistant	Non adherent
cVVC7	P4	*C. albicans*	cVVC	Susceptible	Strongly adherent
cVVC8	P5	*C. albicans*	cVVC	Susceptible	Weakly adherent
cVVC9	P6	*C. albicans*	cVVC	Resistant	Weakly adherent
cVVC10	P7	*C. albicans*	cVVC	Susceptible	Strongly adherent
sVVC1	P8	*C. albicans*	sVVC	Susceptible	Strongly adherent
sVVC2	P9	*C. albicans*	sVVC	Susceptible	Adherent
sVVC3	P10	*C. albicans*	sVVC	Resistant	Strongly adherent
sVVC4	P11	*C. albicans*	sVVC	Susceptible	Weakly adherent
sVVC5	P12	*C. albicans*	sVVC	Resistant	Strongly adherent
sVVC6	P13	*C. albicans*	sVVC	Resistant	Strongly adherent
sVVC7	P14	*C. albicans*	sVVC	Resistant	Strongly adherent
sVVC8	P15	*C. albicans*	sVVC	SDD	Strongly adherent

cVVC: chronic vulvovaginal candidosis; sVVC: sporadic vulvovaginal candidosis. SDD: susceptible dose-dependent.

**Table 2:** Correlation of the results obtained in infection assays (RAW: macrophages, RAW 264.7 cells; Hela: epithelial, HeLa cells) with the results obtained for phenotypic expression of virulence factors. *p*-value according to the Spearman test is shown. PP: proteinase production; CWH: cell wall hydrophobicity; BF: biofilm formation. Adapted with permission from [10]. Copyright license 5320780581818, June 2022. cVVC: chronic vulvovaginal candidosis isolates; sVVC: sporadic vulvovaginal candidosis isolates.

	Internalized (RAW)		Adhered (HeLa)	
	cVVC	sVVC	cVVC	sVVC
**Parameter (*p*-value)**	PP (0.0458)	CWH (0.0216)	No correlation	BF (0.033)

## Supplementary Materials

The following supporting information can be downloaded at: https://www.mdpi.com/article/10.3390/life12060838/s1, Figure S1: Representative result depicting proteinase activity. The enzymatic activity (Pz) was determined by calculating the ratio between the diameters of colony vs. the precipitation zone.
