# Supplementary material for: Vulvovaginal Candida albicans Clinical Isolates’ Resistance to Phagocytosis In-Vitro
Source: Life (Basel). 2022 Jun 4;12(6):838. doi: 10.3390/life12060838 (PMC9225182; doi:10.3390/life12060838)
Supplement: Supplementary file 1 [file life-12-00838-s001.zip › life-1714995-supplementary.pdf]

life

Supplementary Material

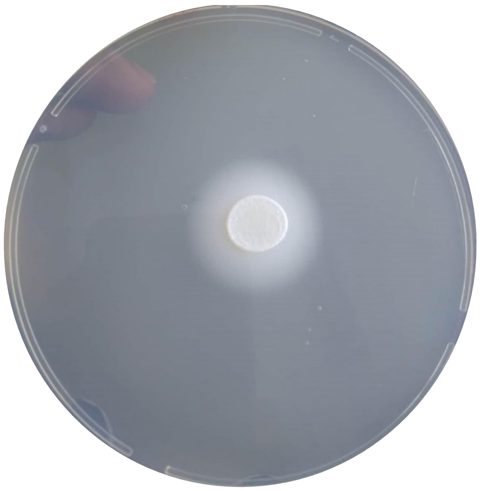

Figure S1. Representative result depicting proteinase activity, indicated by a clear precipitation zone. The enzymatic activity (Pz) was determined by calculating the ratio between the diameters of colony *versus* the precipitation zone.
